# Differential effects on tumor progression by APOBEC3A, APOBEC3B, and APOBEC3H Haplotype I in a breast cancer mouse xenograft model

**DOI:** 10.3389/fgene.2025.1425483

**Published:** 2026-01-28

**Authors:** Milaid Granadillo Rodríguez, Lai Wong, Arzhang Shayeganmehr, Diogo Pellegrina, Frederick S. Vizeacoumar, Franco J. Vizeacoumar, Mohamed Helmy, Linda Chelico

**Affiliations:** 1 Department of Microbiology and Immunology, College of Medicine, University of Saskatchewan, Saskatoon, SK, Canada; 2 Vaccine and Infectious Diseases Organization (VIDO), University of Saskatchewan, Saskatoon, SK, Canada; 3 Department of Pathology and Laboratory Medicine, College of Medicine, University of Saskatchewan, Saskatoon, SK, Canada; 4 Division of Oncology, College of Medicine, University of Saskatchewan, Saskatoon, SK, Canada; 5 Cancer Research Department, Saskatchewan Cancer Agency, Saskatoon, SK, Canada; 6 Vaccinology and Immunotherapeutics Program, School of Public Health, University of Saskatchewan, Saskatoon, SK, Canada; 7 Department of Computer Science, College of Arts and Science, University of Saskatchewan, Saskatoon, SK, Canada

**Keywords:** APOBEC, breast cancer, cancer evolution, mouse xenograft, mutagenesis

## Abstract

**Introduction:**

The APOBEC3 family of cytidine deaminases induces somatic mutations that are highly prevalent in cancers, but the functional consequences remain largely unknown.

**Methods:**

To determine these consequences, we exposed MCF7 tumorigenic breast epithelial cells to APOBEC3A, APOBEC3B or APOBEC3H Haplotype I.

**Results:**

Comparative analysis between cells pre and post ‐APOBEC3 exposure revealed fewer deamination‐dependent γH2AX foci post‐APOBEC3 exposure, despite maintaining APOBEC3 protein expression. In a mouse xenograft model, high expressing, but not low expressing APOBEC3A‐exposed cells caused increased tumor progression. In contrast, high expressing, but not low expressing APOBEC3B‐exposed cells decreased tumor size. APOBEC3H Haplotype I‐exposed cells stochastically increased tumor progression independent of expression levels. Consistent with tumor data, RNA‐seq showed upregulation of tumor enhancing pathways only in cells that enhanced tumor progression.

**Discussion:**

The results indicate that in a breast cancer xenograft model, APOBEC3A and APOBEC3H Haplotype I are more likely to contribute to enhanced tumor progression than APOBEC3B.

## 1 Introduction

Several studies have uncovered a wide range of mutational signatures that reflect the processes that contribute to cancer formation or progression (Alexandrov et al., 2013; Alexandrov et al., 2020; Nik-Zainal et al., 2012; Petljak et al., 2019; Petljak et al., 2022). Deconvolution of single-base substitutions (SBS) revealed that one of the key sources of mutations is the result of apolipoprotein B mRNA editing catalytic subunit-like 3 (APOBEC3)-catalyzed cytosine deaminations in single-stranded (ss) DNA that form promutagenic uracil (Alexandrov et al., 2013; Burns et al., 2013a). These studies uncovered biases of C-to-T (SBS2) as well as C-to-G and C-to-A mutations (SBS13) within preferred trinucleotide motifs, primarily 5′TCA. The C-to-T mutations are the result of replication through unrepaired uracils and C-to-G mutations result from REV1-catalyzed cytosine insertion opposite the abasic site after uracil excision by uracil DNA glycosylase (Petljak et al., 2022; Saini and Gordenin, 2020). APOBEC3 (A3)-catalyzed deaminations occur during DNA replication primarily on the lagging strand or during DNA repair, particularly during Break Induced Replication (BIR) (Saini and Gordenin, 2020; Bhagwat et al., 2016; Seplyarskiy et al., 2016; Hoopes et al., 2016). These APOBEC-associated mutational signatures can be found in more than 50% of cancer genomes and in more than 70% of cancer types (Alexandrov et al., 2020; Bergstrom et al., 2022).

Although A3 enzymes in humans act as potent host restriction factors that can suppress the replication of viruses and retroelements, there is always a potential for off-target deaminations to occur if these enzymes are dysregulated and have access to the nucleus (Adolph et al., 2018; Salter et al., 2017; Cheng et al., 2021; Uriu et al., 2021). APOBEC3A (A3A), APOBEC3B (A3B) and APOBEC3H Haplotype I (A3H Hap I) are the three key enzymes that have access to the nucleus and were identified as contributing to mutagenesis or evolution of multiple cancers, including breast cancer (Burns et al., 2013a; Burns et al., 2013b; Buisson et al., 2019; Cortez et al., 2019; Law et al., 2020; Wormann et al., 2021; Law et al., 2016; Isozaki et al., 2023). Other A3s have also been implicated, including A3G and A3C. Although A3G is primarily cytoplasmic, small amounts of nuclear A3G have been identified to contribute to multiple myeloma and A3G-induced somatic mutations have been observed in bladder cancer (Liu et al., 2023; Talluri et al., 2021). However, A3G has also been found to suppress DNA damage in certain cancers and mouse models suggesting that its effects are cancer type and cancer treatment dependent (Britan-Rosich et al., 2022; Nowarski et al., 2012; Prabhu et al., 2016). A3C that is distributed cell wide has been found to contribute to mutations in pancreatic cancer and pre-leukemia stem cells (Jiang et al., 2021; Qian et al., 2022).

In this study, we focused on breast cancer where A3A, A3B and A3H Hap I mRNA have all been detected. We conducted a parallel study to evaluate their tumorigenic potential since there has been incongruent findings with respect to the roles of A3A and A3B and only a few studies on A3H Hap I (Petljak et al., 2022; Burns et al., 2013a; Cortez et al., 2019; Hix et al., 2020; Starrett et al., 2016). One common finding between A3A and A3B is that they both can cause tumor cell evolution that results in drug resistance in lung cancer (A3A, A3B) and breast cancer (A3B), but a parallel comparison of their effects in breast cells in the absence of chemotherapeutic pressures is not known (Law et al., 2016; Isozaki et al., 2023; Caswell et al., 2024; Garcia et al., 2025). In addition, there has never been direct cause and effect experimental evidence for any of these A3 enzymes enhancing or inhibiting breast tumor formation, only that they contribute to the genetic diversity of breast cancer genomes. A3B was considered to be the main contributor to these APOBEC-associated mutational signatures in breast cancer because of its high expression levels (Burns et al., 2013a; Burns et al., 2013b; Nikkila et al., 2017; Leonard et al., 2013). Some studies have suggested that A3B can drive early mutations in breast and lung cancer, inducing replication stress and chromosomal instability that drives cancer evolution (Venkatesan et al., 2021). A causal relationship for A3B in tumor initiation and evolution in a murine model expressing human A3B showed that A3B can cause accelerated rates of carcinogenesis in some non-mammary tissues (Durfee et al., 2023). However, there has been increasing evidence that A3A plays a more prominent role as it has >100-fold more activity than A3B, can induce large amounts of DNA damage, can promote tumorigenesis in predisposed colon and liver cancer mouse models and although both contribute to recurrent mutations at DNA hairpins, A3A activity is distinct and more predominant (Petljak et al., 2022; Buisson et al., 2019; Cortez et al., 2019; Law et al., 2020; Langenbucher et al., 2021; Wong et al., 2021; Sanchez et al., 2024). When all A3 enzymes were compared in parallel in a murine liver tumor model, only A3A could promote tumorigenesis, however the A3A was also expressed more than the other A3s (Law et al., 2020). Notably, although the mouse models implicate A3A and A3B -induced tumorigenesis, many of the cancers induced in the mouse models are not natural cancers where A3-mutation signatures have been identified, indicating that additional models are needed to establish a cause and effect relationship between A3s and human specific cancers (Alexandrov et al., 2020). A3H Hap I is less studied but has been identified as a contributing factor in somatic mutations in both breast and lung cancer (Hix et al., 2020; Starrett et al., 2016).

Here we established a system to test the functional significance of A3A-, A3B-, and A3H Hap I- induced mutations in a breast cancer model. A past study from our lab established that A3A, A3B, and A3H Hap I have similar abilities to cause DNA damage in MCF10A and MCF7 breast epithelial cells as detected by γH2AX foci, but that their downstream effects on ability to induce cell migration and cell survival after hydroxyurea treatment differed (Granadillo Rodriguez et al., 2023). As a result, we hypothesized that their effects on tumor progression in a mouse xenograft model would also differ. This hypothesis was important to test since multiple studies have determined A3-induced mutation number and that they can cause tumor evolution or therapeutic drug resistance but have been unable to determine if the mutations caused by different APOBEC3 enzymes differ in these functional effects. Only one study tested the functional role of A3B in breast cancer cells (MCF7L) and did not focus on alteration of tumor progression directly, but A3B-induced tamoxifen resistance in breast tumor cells (Law et al., 2016). Here we compared the ability of A3A, A3B, and A3H Hap I to cause tumor evolution.

To compare the ability of the APOBEC3 enzymes to cause tumor evolution we used MCF7 (tumorigenic, luminal A) breast epithelial cell lines that were engineered to stably express doxycycline (dox) inducible A3A, A3B, or A3H Hap I (Granadillo Rodriguez et al., 2023). The stable cell lines were plated into soft agar to maintain anchorage independent growth, a marker of cell transformation and were grown into colonies originating from a single cell. Cells were either exposed or not to the A3s through dox induction. The resulting colonies were then isolated and observed in a mouse xenograft model to test directly if A3 expression changed the ability of MCF7 cells to form tumors in the mammary fat pad of mice. This is the first study conducted in a breast cancer mouse model, a natural cancer where A3-mutation signatures have been identified, that has examined directly the functional significance of A3 expression in the absence of chemotherapeutic agents. We found that A3A in a dose-dependent manner and A3H Hap I, but not A3B, were able to increase tumor size, providing the first direct evidence for a role of A3A and A3H Hap I in enhancing breast tumor progression. In contrast, A3B in a dose-dependent manner decreased tumor size. RNA-seq analysis revealed key pathways that either promoted or inhibited tumor progression. Altogether, the data supports a model in which A3s induce alteration of multiple gene expression pathways to drive tumor progression or regression.

## 2 Materials and methods

### 2.1 Cell culture and generation of stable cell lines expressing A3s

MCF7 polyclonal stable cell lines expressing dox inducible A3A-Flag, A3B-Flag, or A3H Hap I-Flag were generated as described in Granadillo Rodriguez et al., 2023. The following conditions were used to achieve approximately equal steady state protein levels with dox induction: MCF7 A3A-Flag (1 μg/mL dox), MCF7 A3B-Flag (2 μg/mL dox), and MCF7 A3H Hap I-Flag (1 μg/mL dox).

### 2.2 Soft agar assay

MCF7 cell lines transduced to express Flag-tagged A3s were either untreated (Mock, M) or treated (Experimental, E) for 24 h with 1–2 μg/mL dox to achieve similar steady state protein levels (as detailed in Cell culture and generation of stable cell lines expressing A3s). A cell suspension consisting of 30,000 cells in 0.36% top agar (2x EMEM, FBS, 4x MEM vitamin solution) was overlaid in duplicate in 6-well plates that contained 0.61% bottom agar (2x EMEM, FBS, 4x MEM vitamin solution). Plates were maintained for 3 weeks and topped with media (Mock, M) or media containing dox (Experimental, E) every 3 days to prevent desiccation and for experimental conditions, to maintain A3 expression. After 3 weeks, the largest colonies were picked using a 1 mL tip and transferred to a 12-well plate by pipet mixing with culture media.

### 2.3 Immunoblotting

MCF7-derived stable cell lines and soft agar colony cells were treated for 48 h with dox (1–2 μg/mL as detailed in Cell culture and generation of stable cell lines expressing A3s) to induce expression of A3-Flag enzymes. Cells were lysed with 2X Laemmli buffer and 30 µg total protein was used. Flag-tagged enzymes were detected with an anti-Flag mouse antibody (1:1,000, Sigma) and the α-tubulin loading control was detected using an anti-α-tubulin rabbit antibody (1:5,000, Invitrogen). Secondary detection was performed using Licor IRDye antibodies produced in goat (IRDye 680-labeled anti-rabbit and IRDye 800-labeled anti-mouse). Immunoblots were quantified using Image Studio software with normalization of each experimental lane to its respective anti-α-tubulin band.

### 2.4 Immunofluorescence microscopy

MCF7-derived stable cell lines were seeded on glass coverslips and where indicated cells were transfected with 500 ng of a Bacteriophage PBS2 uracil DNA glycosylase inhibitor (UGI) 24 h before dox induction (Feng et al., 2017). Cells were treated for 72 h with 2 μg/mL of dox to induce expression of A3-Flag enzymes. After treatment, cells were permeabilized and stained as previously described (Hix et al., 2020). Data were compiled from two biological replicates. The total cells counted were: A3A (Before), 273; A3A (After), 122; A3B (Before), 229; A3B (After), 511; A3H Hap I (Before), 263; and A3H Hap I (After), 269.

### 2.5 Incucyte cell proliferation

MCF7 cells derived from colonies grown on soft agar, either expressing or not expressing A3s enzymes (A3A, A3B, and A3H Hap I), were cultured to a confluence of approximately 70% in T75 flasks. For the preparation of the 96-well plates for the Live Cell Imager (IncuCyte® S3, Sartorius), the cells were detached from flasks and centrifuged. The pellet was resuspended in fresh media and the cells were counted to seed 4,000 cells in 250 µL of media per well in triplicate. Afterwards, cells were placed in an IncuCyte® S3 live-cell imaging system at 37 °C in a humidified atmosphere containing 5% CO_2_ for a total period of 7 days. Phase contrast images (four images per well) were acquired every 8 h. Percent cell confluence in each well was determined for all time points using the IncuCyte® S3 software.

### 2.6 Mouse xenograft model

All animal protocols were reviewed and approved by the University of Saskatchewan Animal Research Ethics Board (AUP# 20200107). Female NOD/SCID/γ mice (6–8 weeks old) were anaesthetized with isoflurane and supplemented with 0.5 mg, 60-day release 17β-estradiol pellets (Innovative Research of America) 2–3 days prior to injection of MCF7 cells by insertion of the pellets subcutaneously into the nape area with a 10G trocar. When mice were injected with MCF7 breast cancer cells (cell type details in Results), they were again anaesthetized with isoflurane and cells were suspended in PBS (A3A and A3B: 7.5 × 10^6^; A3H Hap I: 0.5 × 10^6^) and mixed 1:1 with Matrigel (Corning) before injection into the mammary fat pad (100 μL total volume). The MCF7-A3H Hap I cells had a faster growth rate than MCF7 -A3A and -A3B cells (Supplementary Figure S1). As a result, less cells were used to maintain similar experimental time courses. Each group contained 5 mice and 2 to 4 independent experiments were carried out for a total of 10–20 mice. Mice were monitored 3 times per week and after 5 weeks for A3H Hap I and 8 weeks for A3A and A3B, mice were euthanized and tumors were excised, fixed in 10% formalin, and weighed. Mice were euthanized for humane reasons when tumors reached the maximum of 1.5 cm in any dimension or due to observed signs of discomfort (weight loss, poor physical appearance, or lack of mobility).

### 2.7 RNA-seq

RNA-seq was performed with two biological replicates from each of the soft agar colonies (mock and experimental). When expanding cells for RNA isolation, the experimental cells were not exposed to dox. The experiment was designed to detect constitutive changes in gene expression from prior A3-induced mutations. The legend for the soft agar colony names is in Supplementary Table S1. RNA preparation and sequencing was performed by the Next-Generation Sequencing Core Lab at the University of Saskatchewan. Total RNA was extracted from cells using the MagMAX mirVana Total RNA Isolation kit (ThermoFisher). Extracted RNA was treated with an additional DNase I treatment (NEB), and purified using the Monarch RNA Cleanup kit (NEB). RNA quality was assessed using the Qubit RNA BR Assay (ThermoFisher), and RNA Screentape (Agilent). Sequencing libraries were generated with 400 ng of input RNA using TruSeq Stranded mRNA Library Prep kit (Illumina). Sequencing libraries were evaluated for quality and quantity using the Qubit dsDNA BR Assay (ThermoFisher) and a D1000 Screentape (Agilent). The barcoded libraries were pooled to equimolar amounts and 75 bp paired-end reads were generated on a NextSeq 550 instrument (Illumina). The sequencing reads were extracted from each run using BaseSpace (Illumina) with default settings. Sequencing adapters and low-quality bases were trimmed using fastp (version 0.23.4) (Chen et al., 2018). The reads were aligned using STAR (version 2.7.9a) (Dobin et al., 2013) and the Human assembly reference (GRCh38.p13). Differential expression between dox induced (experimental) and uninduced (mock) paired samples was assessed using edgeR (Version: 3.36.0) (Robinson and Oshlack, 2010; McCarthy et al., 2012; Chen et al., 2016). RNA-seq data have been deposited at GEO and are available using Accession Number GSE262252.

Signaling and regulatory networks affected by differentially regulated genes were identified using QIAGEN IPA (QIAGEN Inc., https://digitalinsights.qiagen.com/IPA). Exploratory IPA analysis was performed on gene threshold of ↓log fold change↓≥ 3.1.

Comparative analysis of RNA-seq data and breast cancer data from the TCGA database was performed to identify overlapping differentially expressed candidates. Briefly, TCGA patient samples were classified into molecular sub-types using the PAM50 signature (Netanely et al., 2016). By comparing the RSEM (RNA-seq Expectation Maximization) normalized expression of normal breast samples to the Luminal A sub-type patient samples, we identified the genes considered significantly differentially expressed (using the Wilcoxon rank-sum test; p < 0.05). Next, by overlapping these genes with the differentially expressed genes from RNA-seq data, we classified the hits into six categories (1) upregulated in RNA-seq alone (2), downregulated in RNA-seq alone (3), upregulated in both RNA-seq and Luminal A patient data (4), downregulated in both RNA-seq and Luminal A patient data (5), upregulated in RNA-seq but downregulated in Luminal A patient data and (6) downregulated in RNA-seq but upregulated in Luminal A patient data for each of the A3 enzymes (A3A, A3B and A3H Hap I). These overlapping genes were used to generate the volcano plots.

### 2.8 Statistical analysis

Two-tailed t-test was applied for comparisons of two groups, and the results were considered statistically significant at a p-value of ≤0.05. Statistical analyses were performed using SigmaPlot 11.0 software.

## 3 Results

### 3.1 Steady-state A3 protein levels varied in isolated soft agar colonies

**FIGURE 1:**
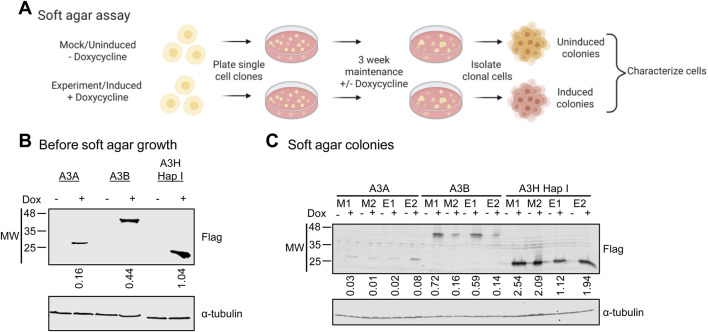
Expression of A3A, A3B and A3H Hap I from isolated MCF7 soft agar colonies. **(A)** MCF7 cells were untreated or pre-exposed to dox for 24 h before plating in soft agar at a dilution enabling growth of colonies from single cells. Cells were maintained in soft agar for 3 weeks under no induction (no dox, Mock, M) or dox induction (dox, Experiment, E) and allowed to form colonies. Colonies were isolated from soft agar and further characterized. **(B)** Immunoblots illustrating expression levels of parental MCF7 stable cell lines expressing A3A-Flag, A3B-Flag or A3H Hap I-Flag (Before soft agar growth). Cells were either untreated (−) or treated (+) with 1–2 μg/mL dox for 48 h before cell lysis and immunoblotting. **(C)** Comparison of expression levels of isolated soft agar MCF7 colonies expressing A3A-Flag, A3B-Flag or A3H Hap I-Flag (Soft agar colonies). The colonies were from mock conditions (M, no dox during 3 weeks soft agar growth) or experimental (E, dox during 3 weeks soft agar growth). Two colonies were tested for each A3 condition to determine if the A3 expression cassette was still functional after growth in soft agar. Cells were not (−) or were (+) treated with 1–2 μg/mL dox for 48 h before cell lysis and immunoblotting. **(B,C)** Immunoblotting was against Flag and α-tubulin. The–and + denotes untreated or treated with dox to induce expression, respectively. Quantification of A3-Flag bands is shown below the Flag blot. Immunoblots were quantified using Image Studio software with normalization of each experimental lane to its respective anti-α-tubulin band.

We plated MCF7 cells stably transduced with an expression cassette for A3A-Flag (A3A), A3B-Flag (A3B), or A3H Hap I-Flag (A3H Hap I) into soft agar (Figure 1A). These cells have low endogenous expression of A3B and A3H Hap I, but the endogenous levels do not cause DNA damage as measured by γH2AX foci (Granadillo Rodriguez et al., 2023). We then exposed those cells to dox (Experiment, E) or no dox (Mock, M) and maintained the cells that were plated in soft agar as single cell clones from the polyclonal A3-expressing parental cells for 3 weeks (Figures 1A,B). Notably, no cell death was observed and the number of colonies formed between the mock and experimental samples was not significantly different for any of the A3 conditions (Granadillo Rodriguez et al., 2023). Since A3-induced mutations are random, we used soft agar to maintain the ability of MCF7 cells to undergo anchorage independent growth, a marker of cell transformation. Media without (Mock, M) or with (Experiment, E) dox was added on top of the agar every 3 days to keep the colonies hydrated and to maintain A3 expression in the experimental conditions (Figure 1A). To investigate the effects of A3-induced mutations after growth in soft agar, two soft agar colonies were isolated from A3A, A3B and A3H Hap I mock and experimental conditions. We determined if the mock and experimental colonies maintained A3 expression by exposing the isolated colonies to dox (+) or no dox (−) for both mock (M) and experimental (E) conditions and then detected the Flag epitope by immunoblotting (Figure 1C). The A3A colonies had lower steady state protein levels in both the mock (M) and experimental (E) conditions than A3B and A3H Hap I, consistent with lower expression in the polyclonal parental cell line (Figures 1B,C). Of the two experimental (E) A3A-expressing colonies, we found by relative comparison, a low (E1) and a high (E2) expressor. The steady state protein levels of the A3B also showed, by relative comparison, low (E2) and high (E1) expressing cells in both mock (M) and experimental (E) samples (Figure 1C). For A3H Hap I, the steady state protein levels were similar in both mock (M) and experimental (E) samples (Figure 1C). A caveat of working with different A3 enzymes is their different expression levels in cell-based experiments, which is not unique to our study (Law et al., 2020). This appears to be due to a combination of protein stability and long-term effects on cells. For example, higher A3-expressing cells may cause cell death, leading to lower expressing cells remaining in the population. Nonetheless, since A3A, A3B, and A3H Hap I have different sensitivities to RNA inhibition and catalytic activities, we determined if they induced similar levels of DNA damage. If the A3-expression produced similar levels of DNA damage, we reasoned that this would be a functional measure of whether there were similar levels of A3 activity in the cells.

### 3.2 A3-induced damage in cells grown in soft agar

**FIGURE 2:**
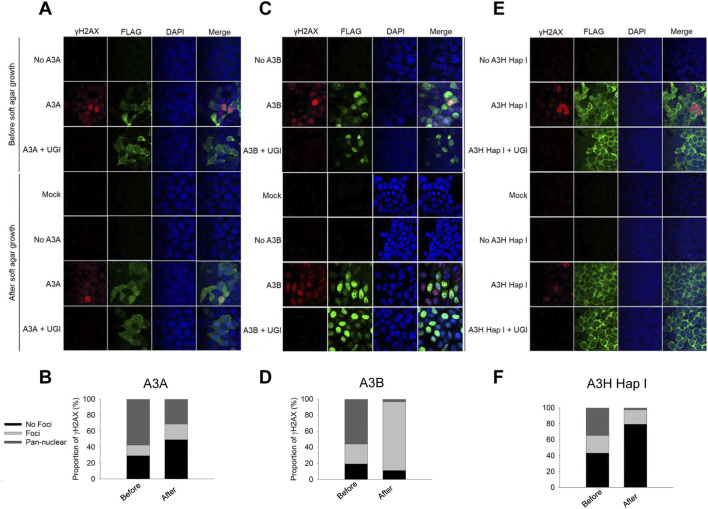
Quantification of γH2AX foci formation in A3 expressing MCF7 cells before and after growth in soft agar. MCF7-derived A3-Flag cell lines before soft agar growth and a colony isolated from soft agar received the following treatments over 72 h before immunofluorescence detection of Flag-tagged A3 (green), γH2AX foci formation (red), and stained nuclei (blue): no dox for 72 h (no A3), treated with dox for 72 h (A3A/A3B/A3H Hap I), or treated with dox for 72 h and transfected with UGI expression plasmid (A3A+ UGI/A3B+ UGI/A3H Hap I + UGI). The mock was an MCF7-derived A3-expressing cell line cultured in soft agar but not grown in the presence of dox and not exposed to dox before immunofluorescence detection. Two independent experiments were conducted and a representative image is shown. The percentage of cells with pan nuclear staining of γH2AX, individual γH2AX foci, or No Foci was determined. **(A,B)** Immunofluorescence microscopy for A3A-Flag and quantification of γH2AX for the high expressing A3A E2 soft agar colony. **(C,D)** Immunofluorescence microscopy for A3B-Flag and quantification of γH2AX for the high expressing A3B E1 soft agar colony. **(E,F)** Immunofluorescence microscopy for A3H Hap I-Flag and quantification of γH2AX for the high expressing A3H Hap I E1 soft agar colony.

We used immunofluorescence to stain for γH2AX foci formation, which is a marker for stalled replication forks and double-strand breaks (DSB) (Rogakou et al., 1998; Ward and Chen, 2001). Parental cells expressing dox-inducible A3 enzymes that were not grown in soft agar (Figure 1B, Before) and cells from A3A high (E2), A3B high (E1), A3H Hap I E1 isolated soft agar colonies (Figure 1C, after) were treated with dox for 72 h to induce A3 expression. For all the parental cells expressing dox-inducible A3 enzymes that were not grown in soft agar (Before), the γH2AX foci formation was similar, confirming that at the beginning of the 3 weeks of A3 expression, cells were exposed to similar A3-induced DNA damage (Figures 2A–F, Before).

For A3A, after growth in soft agar we observed a decrease in the overall percentage of cells that exhibited pan-nuclear staining of γH2AX (Figures 2A,B, 58% of cells before soft agar vs. 31% of cells from the isolated soft agar colony). Additionally, the percentage of A3A-exposed cells having individual γH2AX foci was 2-fold higher compared to A3A before soft agar (Figures 2A,B, 20% of soft agar cells vs. 13% of cells before soft agar). Taken together, these two measurements indicate that A3A-expressing soft agar cells acquired less DNA damage than A3A-expressing parental cells before soft agar. Immunofluorescence demonstrated similar A3A levels in cells before and after soft agar suggesting that cells adapted to decrease A3A activity (Figure 2A).

Similar results were observed with A3B where prior to soft agar growth, we could observe more pan-nuclear γH2AX staining (56%) in contrast to soft agar cells (1%) (Figures 2C,D). Although A3B soft agar cells had fewer cells with pan-nuclear staining than A3A, a population shift was observed where instead 52% of soft agar cells had individual γH2AX foci compared to 24% of before soft agar cells (Figures 2C,D). Again, A3B levels were similar in both before and after soft agar cells, but there was less DNA damage acquired (Figure 2C).

Overall, A3H Hap I had fewer cells with pan-nuclear γH2AX foci than A3A and A3B. Specifically, 34% of A3H Hap I before soft agar cells had pan-nuclear staining whereas A3H Hap I soft agar cells had 2%. A3H Hap I before soft agar cells also had slightly higher proportion of cells with individual γH2AX foci compared to soft agar cells (22% and 18%, respectively) (Figures 2E,F). The change in the ability of A3H Hap I to induce γH2AX foci appeared to be due to less nuclear localization (Figure 2E).

To confirm that A3-catalyzed uracils are the cause of DNA damage, we transfected a bacteriophage protein that is an inhibitor of Uracil DNA Glycosylase (UGI). After UGI transfection, pan-nuclear γH2AX staining disappeared indicating that DNA damage was the result of A3-catalyzed uracils (Figures 2A,C,E).

Altogether, these results demonstrate that the A3-exposed colonies over time decreased the levels of A3-induced DNA damage in the cells. There were distinct activity levels with A3B maintaining the highest activity and A3H Hap I exhibiting the lowest activity, as measured by γH2AX foci. However, the γH2AX foci-inducing activity was similar between A3A, A3B, and A3H Hap I before growth in soft agar.

### 3.3 Long-term A3 expression leads to differential effects on tumor progression

To determine if continuous A3 expression would aid, inhibit, or have no effect on tumor progression we used a mouse xenograft model. We injected cells from two mock and two experimental colonies for each A3. Each colony originated from a single cell clone (Figure 1A). Cells originating from A3A E1 (A3A low), A3A E2 (A3A high), A3B E1 (A3B high), A3B E2 (A3B low) and A3H Hap I E1 and E2 (both A3H Hap I high) soft agar colonies were injected into the mammary fat pads of NOD/SCID/γ mice and compared to their corresponding mock cell population (Figures 1A,C). Since MCF7 parental cells are tumorigenic the experiment aimed to assess whether the A3s could affect tumor progression. These cells were expressing the A3s during the 3-week growth in soft agar, but the mice were not fed any dox to maintain A3 expression. Thus, the experiment would only test if the previously accumulated A3-induced damage had a functional effect. The proliferation rates during normal cell culture of the A3A- and A3B- experimental cells were not altered compared to mock cells, however, the A3H-experimental cells grew slower than the mock cells (Supplementary Figure S1). Notably, the A3H Hap I-expressing cell lines grew faster than the A3A- and A3B- expressing cell lines (Supplementary Figure S1) which necessitated using less MCF7-A3H Hap I cells in the mouse xenograft experiment to enable similar time courses for the experiments with MCF7 -A3A and -A3B cells (see Materials and Methods). There was no effect of dox on the growth rate of parental MCF7 cells (Supplementary Figure S2).

**FIGURE 3:**
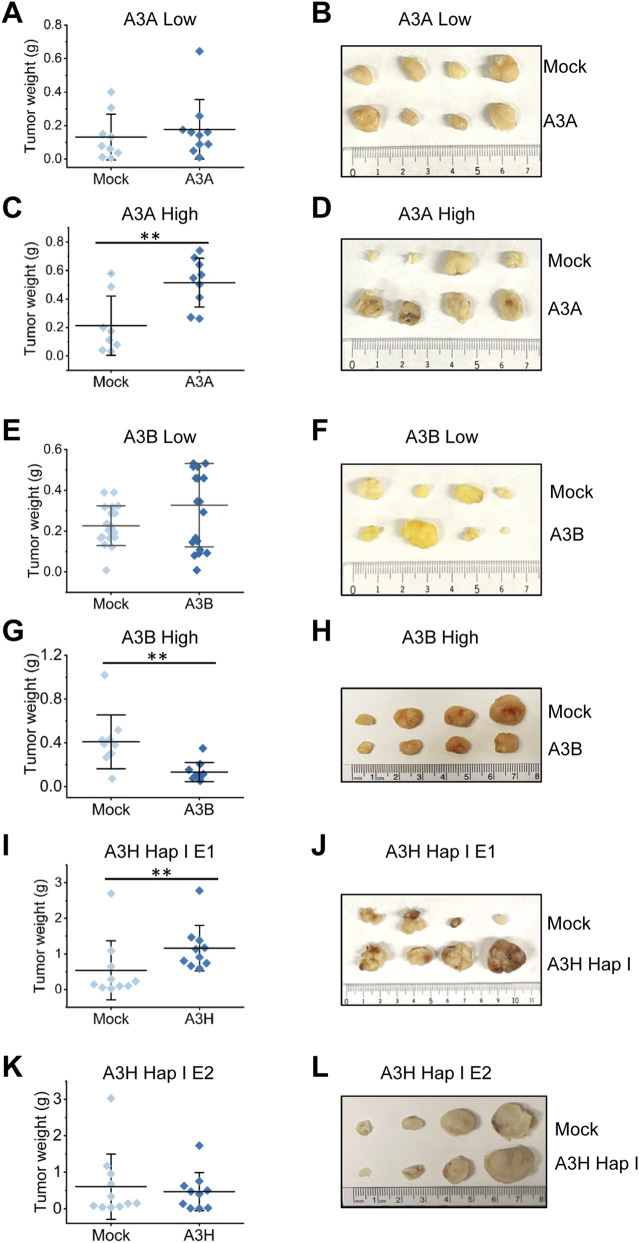
Differential effects of A3A, A3B, and A3H Hap I in a mouse xenograft experiment. Cells from isolated soft agar colonies that were not expressing (Mock, M) or were expressing **(A,B)** low A3A-Flag (E1), **(C,D)** high A3A-Flag (E2), **(E,F)** low A3B-Flag (E2), **(G,H)** high A3B-Flag (E1), **(I,J)** high A3H Hap I-Flag E1, and **(K,L)** high A3H Hap I-Flag E2 were injected into the mammary fat pad of female NOD/SCID/γ mice. Injection of 7.5 × 10^6^ cells (A3A-Flag, A3B-Flag) or 0.5 × 10^6^ cells (A3H Hap I-Flag) were performed 2–3 days post 17β-estradiol pellet implantation. Tumors were excised 5 weeks (A3H Hap I) or 8 weeks (A3A and A3B) post injection and weighed. Representative images of tumor sizes from one experiment are shown. At least two independent experiments were conducted with five mice per experiment. The statistical differences were determined by t-test where *p ≤ 0.05, **p ≤ 0.01, and ***p < 0.001.

The mouse xenograft experiments yielded different results for each A3 and each colony of A3-exposed soft agar cells. No differences were observed in tumor progression in the mice injected with low A3A-exposed colony (Figures 3A,B, 5 mice per group in two independent biological experiments (n = 10 mice total per group)). In contrast, statistically significant larger tumor sizes were observed for the high A3A-exposed colony (Figures 3C,D, 5 mice per group in two independent biological experiments (n = 10 mice total per group)). The low A3B-exposed colony had 2-fold more A3B steady state protein levels upon dox induction than the high A3A-exposed colony (Figure 1B). Although the low A3B-exposed colony compared to the mock did show a general trend in enhanced tumor progression, it was not statistically significant, suggesting that the effect of A3B on cells is different than A3A (Figures 3E,F, 5 mice per group in four independent biological experiments (n = 20 mice total per group)). Consistent with A3B having a different effect than A3A, the high A3B-exposed colony significantly decreased tumor progression (Figures 3G,H, 5 mice per group in two independent biological experiments (n = 10 mice total per group)). Notably, the A3A high (E1) and A3B high (E2) colonies did induce similar levels of γH2AX foci at the beginning of the 3 weeks dox induction, suggesting that despite different steady-state protein levels, their activity levels within these cell clones were similar (Figures 1B,C, 2A–D). The A3H Hap I-exposed colonies both had similar protein expression levels (Figure 1B). However, we observed different results for each colony. The A3H Hap I-exposed E1 colony showed statistically significant larger tumors, but the A3H Hap I-exposed E2 colony showed no significant difference from the mock (Figures 3I–L, 5 mice per group in two independent biological experiments (n = 10 mice total per group)).

### 3.4 RNA-seq reveals tumorigenic changes to transcriptome in soft agar colony cells

To understand how the A3-exposed soft agar colonies could have different effects, we investigated the changes in gene expression. We compared gene expression by RNA-seq in the A3-exposed compared to mock soft agar colonies. The A3-exposed and mock soft agar colonies were expanded for RNA isolation in the absence of dox treatment, thus identifying changes made to the cells’ DNA that stably altered the RNA profile during the growth in soft agar. This analysis facilitates the understanding of the cause-and-effect relationship between high or low A3 expression, rather than merely correlating with the number of mutations. The functional significance of these changes was interpreted using ingenuity pathway analysis (IPA). For the IPA canonical pathways, we examined pathways that were significant (-log (p-value) >1.3) and then from those pathways we determined whether there was a general trend of up (>2 z-score) or down regulation (<-2 z-score) in the pathway. The data were plotted as a bubble plot with the -log (p-value) and the z-score. (the reference to axes was reversed) on the x- and y-axes, respectively, and the bubble representing the percentage of genes in the pathway that were significantly altered (Ratio).

**FIGURE 4:**
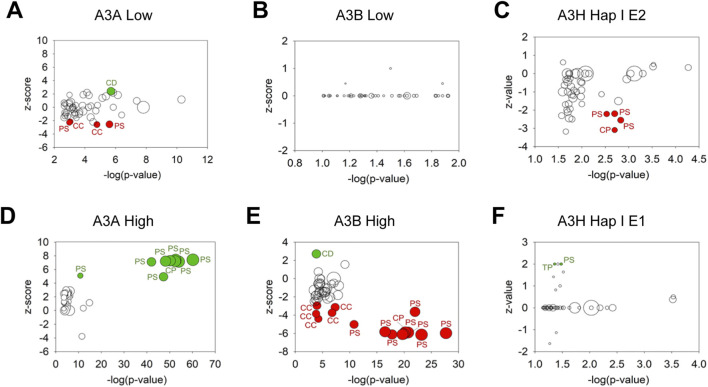
RNA-seq analysis revealed multiple pathways altered after A3 expression. The changes in gene expression of A3-exposed cells compared to mock cells was determined. The statistically significant changes were then used for IPA analysis. IPA identified changes in canonical gene expression pathways that are shown as a function of the z-value (>2, upregulated pathway; <-2, downregulated pathway), -log (p-value), and ratio (bubble, percentage of genes in pathway identified). The pathways identified for **(A–C)** colonies that did not alter tumor progression from the mock are shown in **(A)** A3A Low (E1), **(B)** A3B Low (E2), **(C)** and A3H Hap I E2 and pathways identified for **(D–F)** colonies that did alter tumor progression from the mock are shown in **(D)** A3A high (E2), **(E)** A3B high (E1), **(F)** and A3H Hap I E1.

The use of IPA enabled general trends to be identified. First, for the A3A and A3B -exposed soft agar colonies that did not alter tumor progression (A3A low, A3B low), the gene expression pathways were usually not significantly altered as indicated by few pathways that were outside of the <-2 and >2 range (Figures 4A,B). In all but one case, for A3A low and A3B low, the significantly altered pathways were predominantly downregulated (Figures 4A,B). In contrast for the A3A and A3B -exposed soft agar colonies that did alter tumor progression (A3A high, A3B high), the gene expression pathways were more significantly altered as indicated by more pathways that were outside of the <-2 and >2 range (Figures 4D,E). The A3A high that increased tumor progression had increases in multiple gene expression pathways, but the A3B high that decreased tumor progression had decreases in multiple gene expression pathways. An exception was observed with the A3H Hap I E1 and E2 colonies that appeared to have minimal effects on altering gene expression pathways (Figures 4C,F). However, while the A3H Hap I-exposed soft agar colony that enhanced tumor progression (E1) showed an increase in gene expression pathways, the colony with no effect on tumor progression (E2) exhibited decreases in gene expression pathways (Figures 4C,F).

The identities of the altered gene expression pathways demonstrated clear trends. The A3-exposed soft agar colonies that did not alter tumor progression had either no change in gene expression pathways from the mock (A3B low) or downregulation of pathways involved in protein synthesis (PS), cell cycle (CC) or cell proliferation (CP) (Figures 4A–C and Supplementary Figures S1, S2). For the A3A low condition there was also upregulation of a cell death (CD) gene expression pathway (Figure 4A and Supplementary Figures S1, S2, Granzyme A Signaling). For the A3-exposed soft agar colonies that enhanced tumor progression there was upregulation of pathways involved in PS and CP (A3A high) and PS and tumor progression (TP) (A3H E1) (Figures 4D-F and Supplementary Materials 1,2). For A3B high that had the opposite effect on tumor progression from A3A and A3H Hap I, there were opposite effects where we observed downregulation of CC, PS, and CP pathways and upregulation of a CD pathway (Figure 4E and Supplementary Materials 1,2). Notably, the A3A high and A3B high that had opposing effects on tumor size altered the same PS pathways in opposite ways (down versus upregulation). The PS pathways that appeared to be most essential in tumor progression with differential effects from A3A and A3B were: Eukaryotic Translation (Elongation, Termination, Initiation), EIF2 signaling, Selenamino acid metabolism, Nonsense-Mediated decay, SRP-dependent cotranslational protein targeting to membrane, Major pathway of rRNA processing in the nucleolus and cytosol, and Processing of Capped Intron-Containing Pre-mRNA (Supplementary Materials 1,2). However, A3A high and A3B high also differentially regulated the CP pathway of Response of EIF2AK4 (GCN2) to amino acid deficiency and CC pathway of Mitotic G2-G2/M phases (Supplementary Materials 1,2). The A3B high colony also showed downregulation of multiple other CC pathways (Cell Cycle Checkpoints, Mitotic Metaphase and Anaphase, Mitotic Prometaphase, Synthesis of DNA, Supplementary Materials 1,2). A3H Hap I E1 and E2 colonies had different effects from each other and A3A and A3B on gene expression. The A3H Hap I E1 that increased tumor size showed upregulated genes involved in Electron transport, ATP synthesis, and heat production by uncoupling proteins (PS) and MSP-RON Signaling in Cancer Cells Pathway (TP) (Supplementary Materials 1,2). In contrast, A3H Hap I E2 that had no effect on tumor size showed downregulated pathways in PS relating to Processing of Capped Intron-Containing Pre-mRNA, MicroRNA Biogenesis Signaling, RNA Polymerase II Transcription and in CP relating to RHO GTPase cycle (Supplementary Materials 1,2).

We used The Cancer Genome Atlas (TCGA) to determine if any of the changes in the MCF7 cell lines corresponded to changes found in breast cancers of the Luminal A subtype to which MCF7 cells belong (Comsa et al., 2015). There was on average ∼13% overlap with up or 9% overlap with downregulated genes in cancer genomes (Supplementary Figure S3 and Supplementary Materials 3). These data illustrate the heterogeneity that can develop in cancer genomes. Some of the genes overlapped with the altered gene regulation pathways identified by IPA, especially for the A3A high colony (Supplementary Materials 2). That the most overlap occurred with the A3A high colony is consistent with A3B not causing enhanced tumor progression and A3H having the least effect on gene expression changes.

## 4 Discussion

Evidence of the involvement of A3s in cancer evolution has largely been based on mutational signatures and mRNA expression data (Alexandrov et al., 2013; Alexandrov et al., 2020; Nik-Zainal et al., 2012; Petljak et al., 2019; Petljak et al., 2022). Recently mouse models have shown that A3A and A3B can initiate tumor formation and contribute to tumor evolution, but none examined breast cancer where A3-induced mutations are highly prevalent (Alexandrov et al., 2020; Law et al., 2020; Durfee et al., 2023). Here we directly tested the effect of the A3 enzymes reported to contribute to breast cancer by using a NOD/SCID/γ mouse xenograft model. Cells previously exposed to A3 enzymes for 3 weeks prior to injection into mice were used to test the effect of preexisting DNA damage. Our results indicated that expression of A3A and A3H Hap I can lead to larger tumor size. These are the first findings to show A3A-enhanced tumor progression in breast cancer and the first data to show A3H Hap I-induced tumor progression in any cell type. We did not observe that A3B could enhance tumor progression in our model but instead decreased tumor progression. These contrasting effects between A3B and A3A in tumor progression correlated to opposing changes in gene expression. A3H Hap I had unique effects on gene expression compared to A3A and A3B.

Few previous studies have directly tested whether A3 expression can drive tumorigenesis. Law et al. used a panel of A3 enzymes and compared their ability to induce tumor formation in murine livers (Law et al., 2020). Only A3A expression was reported to promote tumorigenesis with a caveat being that A3A was also expressed more than the other A3s (Law et al., 2020). In contrast, our mouse model used single cell clones with high and low expression of A3A, A3B, and two clones with equal expression of A3H Hap I. The data showed different dose-dependent effects for both A3A and A3B. As the A3A steady-state levels were increased, so did tumor size, consistent with Law et al. (2020). However, we did not find an increase in tumor size with an increase of A3B steady-state levels in cells. Rather, A3B at lower levels had no effect and A3B at higher levels decreased tumor size. Decreases in tumor size in the presence of A3B has also been observed in EGFR mutant non-small-cell lung cancer (Caswell et al., 2024). Nonetheless, in the presence of targeted therapies, A3B can also lead to therapy resistance in breast and lung cancer (Law et al., 2016; Caswell et al., 2024; Garcia et al., 2025). Law et al. using MCF7L cells and saw no effect of A3B on tumor size, similar to the A3B low colony in our study (Law et al., 2016). However, Law et al. used different experimental conditions and did not study different A3B levels in cells (Law et al., 2016). Thus, our study uniquely observed in MCF7 cells that higher A3B expression causes a decrease in tumor size.

Despite A3B being considered for many years as the major A3 in breast cancer because of its high expression, several studies have provided evidence that it may not play as prominent of a role in tumor development as initially thought (Burns et al., 2013a; Cortez et al., 2019; Law et al., 2020; Chan et al., 2015). Consistent with our study A3B has been found by others to have non-tumorigenic roles and be involved in the generation of neoepitopes and at least for human ovarian cancer high A3B expression correlated with a positive prognosis (Driscoll et al., 2020; DiMarco et al., 2022; Long et al., 2023). Studies have reported that APOBEC-induced mutations within the A3B-preferred 5′RTCA motif appeared less frequently than the A3A-preferred 5′YTCA motif in breast cancers and only modestly correlated with expression whereas A3A had a strong correlation (Cortez et al., 2019; Chan et al., 2015). Our data with MCF7 cells is in agreement with a previous study that found A3B-induced mutagenesis does not initially contribute to EGFR mutant non-small-cell lung cancer and this may be due to A3B downregulating tumor progression gene expression pathways and upregulating causing cell death gene expression pathways (Caswell et al., 2024). Nonetheless, A3B may have a tumorigenic role in other cancers as shown by a mouse model that expresses human A3B in multiple tissues and cell line-based studies that suggested A3B may also act in concert with A3A (Petljak et al., 2022; Durfee et al., 2023; Carpenter et al., 2023). The A3B-expressing mouse model showed increased lymphoid and liver tumor formation, but these are not natural cancers where the A3 mutation signature is found (Alexandrov et al., 2020; Durfee et al., 2023). All data to date and our study show that A3B contributes to tumor heterogeneity, but there must be tissue differences that decrease the tumorigenic potential of A3B in at least human breast and lung cells, in the absence of chemotherapeutic treatments (Caswell et al., 2024; Venkatesan et al., 2021; Durfee et al., 2023; Schreurs et al., 2025).

Importantly our data resulted in the novel observation that A3H Hap I can promote tumor evolution. A3H Hap I is less studied, but there is evidence of A3H Hap I-induced mutations in cancer (Starrett et al., 2016). After deletion of A3A and A3B, Petljak et al. did observe evidence of a remaining cytidine deaminase in cancer cell lines, although the specific A3 was not identified (Petljak et al., 2022). Starrett et al. observed a mutation signature in *A3B* ^*−/-*^ donors in the absence of A3A mRNA that was attributed to A3H Hap I (Starrett et al., 2016). In agreement with our results, some studies have found no impact of A3H Hap I (Law et al., 2020; Carpenter et al., 2023). Although A3H Hap I was initially characterized to be unstable in cells, later research showed it is rapidly targeted for ubiquitination, but not inherently unstable (Chesarino and Emerman, 2020). In cancer cells, ubiquitination pathways can be less functional and may lead to higher amounts of A3H Hap I. Although we did observe effects of A3H Hap I E1 on tumor size, this was not consistent with A3H Hap I E2, despite similar expression levels. The concentration independent effects of A3H Hap I in our study may reflect the stochastic nature of A3 activity and induced mutagenesis or the variable steady state levels of A3H Hap I (Chesarino and Emerman, 2020). Although we found that A3A, A3B, and A3H Hap I induced similar amounts of γH2AX foci before soft agar in MCF7 cells, the resulting mutations may differ. A3A has been found to induce the highest number of mutations, followed by A3B, and they appear to cause mutations in different hot spots (Petljak et al., 2022; Buisson et al., 2019; Sanchez et al., 2024). Less information is known about the ability of A3H Hap I to cause mutations. Nonetheless, these data provide an explanation for the different results obtained between the three A3 enzymes.

Despite A3A, A3B, and A3H Hap I all being implicated in breast cancer mutagenesis, their direct impact was not previously tested. Here we determined the effect of A3 expression on a mouse xenograft with MCF7 breast epithelial cells. Our data support more prominent roles for A3A and A3H Hap I, rather than A3B in enhancing breast tumor progression and that A3B may be more likely to decrease tumor progression in the absence of chemotherapeutic agents. Despite A3B being characterized to cause more mutations than A3H Hap I, the different target site specificities and stability in cells may result in different functional outcomes that do not correlate with mutation numbers (Petljak et al., 2022; Starrett et al., 2016). Transcriptome data underscores the diverse impact of A3 enzymes on gene expression and that the impact of each was unique. Altogether the data highlight the importance of characterizing functional changes induced by A3 enzymes, rather than focusing solely on mutations. Finally, the data emphasize that multiple A3s should be the focus of breast cancer studies since A3A, A3B, and A3H Hap I each had unique effects.

## Data Availability

The datasets presented in this study can be found in online repositories. The names of the repository/repositories and accession number(s) can be found in the article/Supplementary Material.
