# Supplementary material for: Differential effects on tumor progression by APOBEC3A, APOBEC3B, and APOBEC3H Haplotype I in a breast cancer mouse xenograft model
Source: Front Genet. 2026 Jan 28;16:1425483. doi: 10.3389/fgene.2025.1425483 (PMC12890242; doi:10.3389/fgene.2025.1425483)
Supplement: Supplementary file 1 [file Table1.pdf]

Supplementary Table 1. Legend for the soft agar colony names.

Soft Agar Colony Names	Name in Results
A3A U1/A3A I4	A3A Low, M1/E1
A3A U6/A3A I5	A3A High, M2/E2
A3B U2/A3B I5	A3B Low, M2/E2
A3B U1/A3B I2	A3B High, M1/E1
A3H U1/A3H I4, High-1	A3H Hap I M1/E1
A3H U2/A3H I1, High-2	A3H Hap I M2/E2
