# Supplementary material for: Differential effects on tumor progression by APOBEC3A, APOBEC3B, and APOBEC3H Haplotype I in a breast cancer mouse xenograft model
Source: Front Genet. 2026 Jan 28;16:1425483. doi: 10.3389/fgene.2025.1425483 (PMC12890242; doi:10.3389/fgene.2025.1425483)
Supplement: Supplementary file 2 [file Presentation1.pdf]

Supplementary Information

Differential effects on tumor growth by APOBEC3A, APOBEC3B, and APOBEC3H Haplotype I in a breast cancer mouse xenograft model

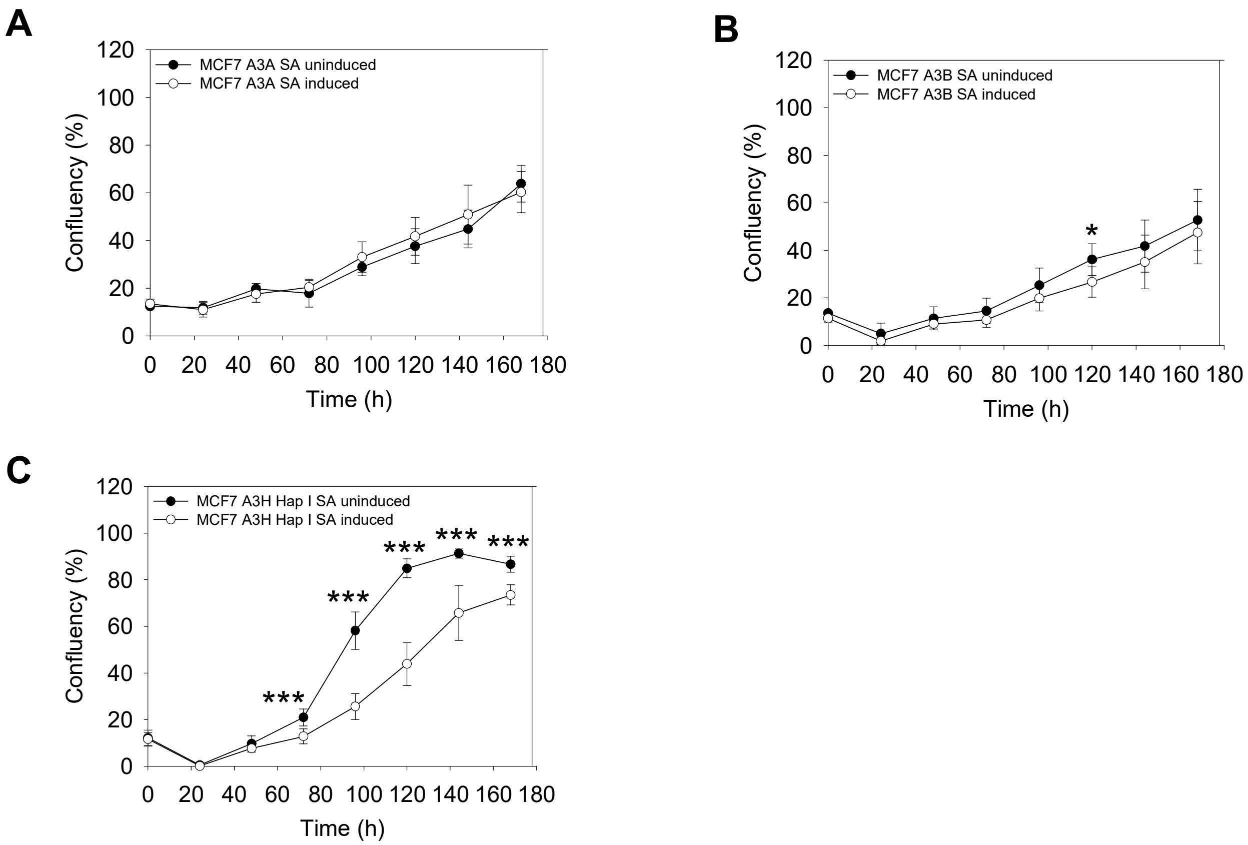

Supplementary Figure 1. Cell proliferation of isolated soft agar colonies. Cell proliferation was measured using live cell imager IncuCyte® S3 over a span of 168 h for cells that expressed A) A3A B) A3B and C) A3H Hap I expressed as percent cell confluence. The growth was compared to the mock cell population. Error bars indicate the S.D. from the mean for three biologically independent replicates. A t-test determined significantly different values that are marked as * $p \leq 0.05$, ** $p \leq 0.01$, and *** $p \leq 0.001$.

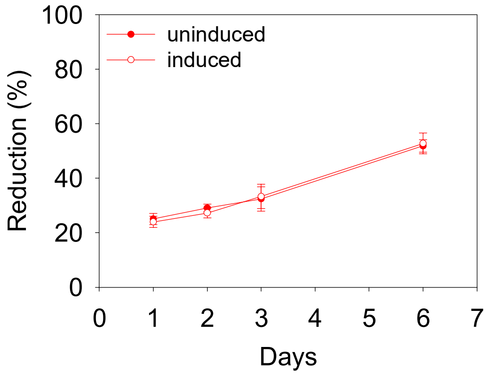

Supplementary Figure 2. Doxycycline does not have an effect on cell proliferation of parental MCF7 cells. For the assay, 4 x 10^3 MCF7-derived stable cells were seeded in triplicate in 96-well plates and were either treated with dox for 6 days with 2 µg/mL dox (induced) or left untreated (uninduced). Cell proliferation was monitored for 6 days and 10% of Alamar Blue (AB) dye (Invitrogen) was added on day 1, 2, 3, and 6 followed by a 4 h incubation at 37°C. Percentage of AB reduction was calculated using the following formula after the absorbance was measured at 570 and 600 nm: reduction (%) = [(117.216) A570- (80.586) A600] / [(155.677) A'600- (14.652) A'570] x 100% [61] Where 117.216 and 80.586 are the molar extinction coefficient of AB in the oxidized form at 600 and 570 nm, respectively; 14.652 and 155.677 are the molar extinction coefficient of AB in the reduced form at 600 and 570 nm, respectively; A600 and A570 are the absorbance of test wells at 600 and 570 nm, respectively; A'600 and A'570 are the absorbance of negative control wells at 600 and 570 nm.

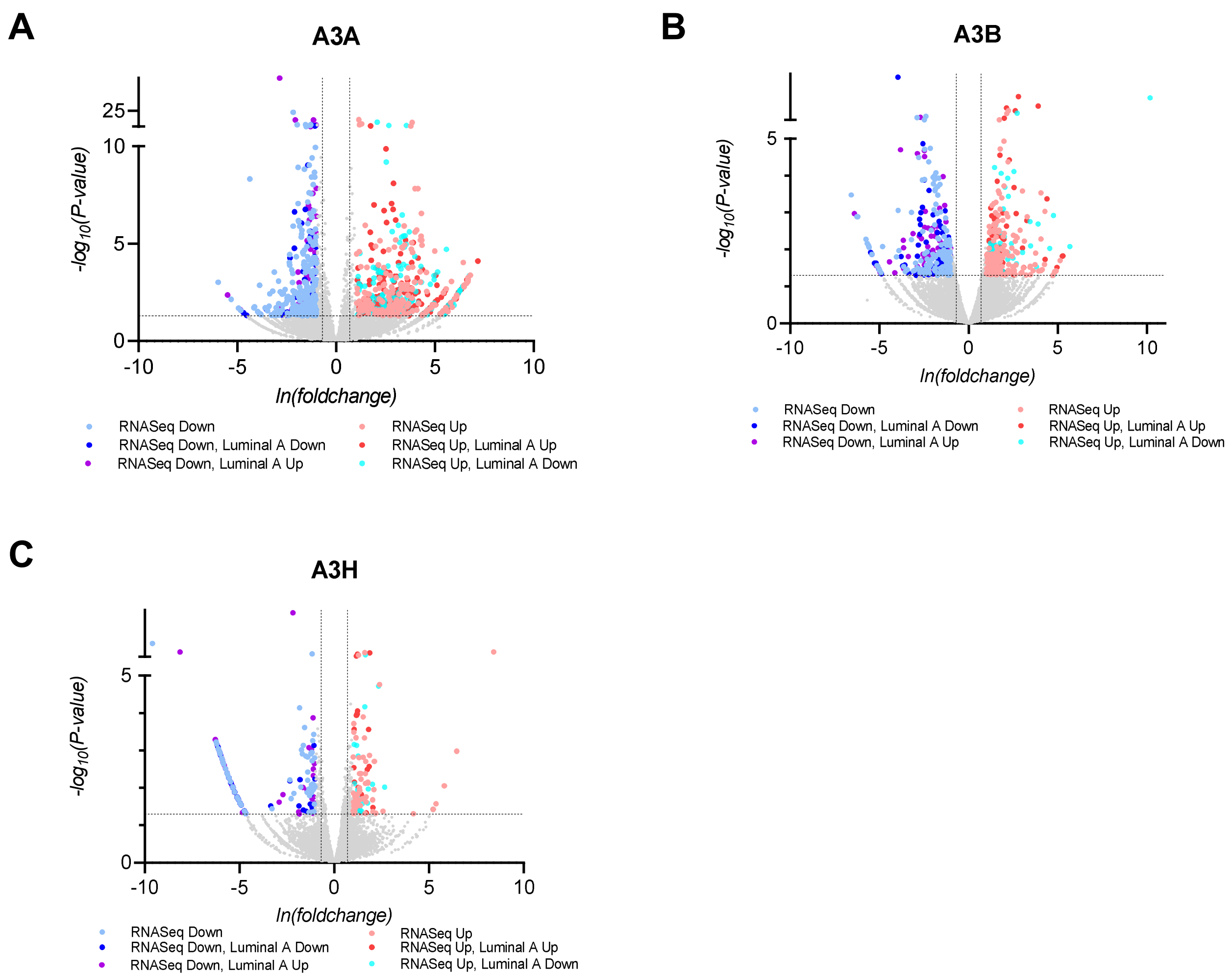

Supplementary Figure 3. RNAseq identified differential gene expression in isolated soft agar colonies. The changes in gene expression of A3-exposed cells from mock cells was determined. The gene expression changes were then further compared to gene expression changes in Luminal A breast cancers in the TCGA. Volcano plots illustrating the comparison of these two RNAseq sets is shown for (A) A3A high-, (B) A3B high-, and (C) A3H Hap I (A3H) E1- exposed cells. Comparisons shown are: mRNA are down regulated compared to the mock, down regulated in both RNAseq cells and Luminal A breast cancers, down regulated in RNAseq, but up regulated in Luminal A breast cancers, up regulated compared to the mock, up regulated in both RNAseq cells and Luminal A breast cancers, and up regulated in RNAseq, but down regulated in Luminal A breast cancers. For overlapping data sets (both upregulated or down regulated) we found the number of common genes from the total were: A3A, 273 mRNA were upregulated (18% overlap) and 60 mRNA were down-regulated (4% overlap) in both RNAseq data and TCGA Luminal A datasets; A3B, 86 were up-regulated (11% overlap) and 78 were down-regulated (10% overlap) in both RNAseq data and TCGA Luminal A datasets; A3H Hap I, 55 were up-regulated (9% overlap) and 83 were down-regulated (13% overlap) in both RNAseq data and TCGA Luminal A dataset.
